# Native Microalgae-Bacteria Consortia: A Sustainable Approach for Effective Urban Wastewater Bioremediation and Disinfection

**DOI:** 10.3390/microorganisms12071421

**Published:** 2024-07-12

**Authors:** Joana F. Sousa, Helena M. Amaro, Sara Ribeirinho-Soares, Ana F. Esteves, Eva M. Salgado, Olga C. Nunes, José C. M. Pires

**Affiliations:** 1 LEPABE—Laboratory for Process Engineering, Environment, Biotechnology and Energy, Faculty of Engineering, University of Porto, Rua Dr. Roberto Frias, 4200-465 Porto, Portugal; up201806415@edu.fe.up.pt (J.F.S.); lena.amaro@gmail.com (H.M.A.); up201305551@edu.fe.up.pt (S.R.-S.); up201405959@edu.fe.up.pt (A.F.E.); up201606419@edu.fe.up.pt (E.M.S.); opnunes@fe.up.pt (O.C.N.); 2 ALiCE—Associate Laboratory in Chemical Engineering, Faculty of Engineering, University of Porto, Rua Dr. Roberto Frias, 4200-465 Porto, Portugal; 3 LSRE-LCM—Laboratory of Separation and Reaction Engineering-Laboratory of Catalysis and Materials, Faculty of Engineering, University of Porto, Rua Dr. Roberto Frias, 4200-465 Porto, Portugal

**Keywords:** antibiotic resistance, *bla*_TEM_, effluent treatment, *Escherichia coli*, *intI*1, nutrient recovery, *sul*1

## Abstract

Urban wastewater is a significant by-product of human activities. Conventional urban wastewater treatment plants have limitations in their treatment, mainly concerning the low removal efficiency of conventional and emerging contaminants. Discharged wastewater also contains harmful microorganisms, posing risks to public health, especially by spreading antibiotic-resistant bacteria and genes. Therefore, this study assesses the potential of a native microalgae-bacteria system (MBS) for urban wastewater bioremediation and disinfection, targeting NH_4_^+^-N and PO_4_^3−^-P removal, coliform reduction, and antibiotic resistance gene mitigation. The MBS showed promising results, including a high specific growth rate (0.651 ± 0.155 d^−1^) and a significant average removal rate of NH_4_^+^-N and PO_4_^3−^-P (9.05 ± 1.24 mg L^−1^ d^−1^ and 0.79 ± 0.06 mg L^−1^ d^−1^, respectively). Microalgae-induced pH increase rapidly reduces coliforms (*r* > 0.9), including *Escherichia coli*, within 3 to 6 days. Notably, the prevalence of *intI*1 and the antibiotic resistance genes *sul*1 and *bla*_TEM_ are significantly diminished, presenting the MBS as a sustainable approach for tertiary wastewater treatment to combat eutrophication and reduce waterborne disease risks and antibiotic resistance spread.

## 1. Introduction

The world faces escalating challenges due to continuous global population growth and economic development, namely pollution and resource scarcity [1]. In recent decades, anthropogenic activities, such as urbanisation, industrialisation, and agriculture, have led to substantial wastewater generation. Each year, the annual worldwide municipal wastewater production reached a staggering 380 × 10^9^ m^3^, with an unsatisfactory fraction undergoing treatment before discharge into natural bodies of water [2,3]. Uncontrolled wastewater disposal poses severe environmental pollution and human health risks. Eutrophication, characterised by excess nitrogen and phosphorus compounds in water bodies, is a significant consequence of inadequate wastewater discharge. It leads to harmful algal blooms, oxygen depletion due to the microbial decomposition of excess biomass, and the loss of essential species, resulting in the overall degradation of freshwater ecosystems [4]. Domestic sewage introduces waterborne pathogens into the environment, causing diseases such as diarrhoea, gastroenteritis, dysentery, meningitis, and cholera through contaminated water consumption or contact. Additionally, wastewater contributes to the environmental dissemination of antibiotic-resistant bacteria and antibiotic-resistant genes (ARGs), a growing global threat highlighted by the World Health Organisation (WHO), which warns of ten million annual deaths by 2050 if immediate action is not taken [5]. Furthermore, wastewater pollution includes heavy metals, xenobiotics, and microplastics, further endangering the food chain and the foundation of human life [6]. In light of these challenges, addressing wastewater management and pollution mitigation is crucial for a sustainable future.

Recently, microalgae have emerged as promising agents for bioremediation, particularly in tertiary wastewater treatment. These microscopic organisms exhibit remarkable metabolic flexibility, produce minimal to no toxic by-products, and offer the added advantage of yielding biomass that can be further converted into value-added products such as biofertilisers or biofuels [7,8,9]. Microalgal systems encompass communities of microalgal cells, which can comprise single microalgal species (monoculture), multiple microalgae species (polyculture), or a combination of microalgae and bacteria (microalgae-bacteria consortia). In recent years, research in microalgal systems for wastewater treatment has explored various scenarios involving real and synthetic wastewater at laboratory or pilot scales [10,11,12]. Initial investigations primarily focused on laboratory-scale experiments using synthetic or sterilised wastewater [13,14]. These studies often centre on microalgae monocultures or the identification of microalgae-bacteria consortia. However, maintaining a monoculture of microalgae in wastewater treatment applications becomes impractical due to the inherent diversity of microorganisms in wastewater. Using microalgal polycultures offers a more realistic and practical approach, providing increased robustness, scalability, and feasibility for bioremediation. This is attributed to the diverse nutritional requirements and adaptability of different microalgal species within the polyculture. Furthermore, microalgal polycultures significantly enhance nutrient uptake efficiency, making them a compelling choice for wastewater treatment [7,8].

Microalgae-bacteria systems (MBSs) provide efficient nutrient removal from wastewater from different sources and can even promote their disinfection. Although these systems were not developed for the latter purpose, their operational characteristics generate hostile conditions for many pathogens. This phenomenon arises from a complex interaction of various abiotic factors, including pH, dissolved oxygen concentration, light exposure, photoperiod, CO_2_ levels, and hydraulic retention time [7]. Removing pathogens is a complex process across different bacterial strains, owing to their diverse cellular structures, metabolic pathways, and cell regeneration processes [7,15]. To identify the key parameters influencing *E. coli* and enterococci disinfection, Ouali et al. [16] tested different values of irradiation (0–25 W m^−2^), pH (5.16–12.07), and dissolved oxygen concentration (DO, 1.2–8.9 mg L^−1^) in a pilot-scale maturation pond. The results showed that *E. coli* removal was closely dependent on irradiation, DO, and pH (significantly increasing when the pH was above 8.5). Enterococci removal was mainly driven by irradiation and DO as well. Additional mechanisms of pathogen removal by microalgae in wastewater treatment systems have been suggested [7]: (i) production of humic substances; (ii) secretion of antimicrobial metabolites and toxins; and (iii) attachment of bacteria to microalgal cells followed by sedimentation. Khuda et al. [17] demonstrated that humic acids (HAs) exhibited substantial antibacterial activity against *Salmonella typhi*, *Pseudomonas aeruginosa*, and *E. coli*, with minimum inhibitory concentration values of 0.82 mg mL^−1^, 0.87 mg mL^−1^, and 0.79 mg mL^−1^, respectively, in addition to displaying significant antifungal activity. While these mechanisms offer insights into the potential of microalgae-based wastewater treatment systems, it is important to note that the precise mechanism through which microalgal growth reduces ARGs still needs to be fully understood. However, several contributing factors have been proposed: (i) microalgal growth can induce changes in the bacterial community within the wastewater, potentially impacting the prevalence of ARGs; and (ii) microalgae can remove or degrade certain antibiotics through processes namely adsorption, biodegradation, and photodegradation—this could reduce the selective pressure for antibiotic resistance in bacteria [18,19]. Tang et al. [20] observed that a consortium of microalgae (*Chlorella vulgaris*) and bacteria (*Bacillus licheniformis*) enhanced the reduction of extracellular deoxyribonucleic acid (DNA), measured by the abundance of the sulphonamide resistance gene *sul*1 carried in a plasmid added to the culture. They proposed that exogenous plasmids could be adsorbed to the surface of the consortium’s cells, with extracellular polymeric substances (EPS) increasing adsorption sites. In addition, microalgal cells seem able to endocytose exogenous ARG-carrying plasmids [20]. However, it is crucial to recognise that these findings have primarily been demonstrated in controlled laboratory settings with synthetic media and controlled microbial populations. The complexity of real urban wastewater (UWW) composition introduces additional variables and challenges. Therefore, while these mechanisms hold promise, their applicability and efficacy in real-world UWW scenarios may vary. Further research is needed to understand and optimise these mechanisms in practical wastewater treatment applications.

Even though numerous studies have been conducted using microalgal systems to remove conventional pollutants and, to a lesser extent, pathogens and ARGs, there remains a significant gap. None of these studies have comprehensively investigated the simultaneous removal of these diverse parameters and the underlying biotic and abiotic factors contributing to their elimination. Hence, this study aims to bridge this crucial knowledge gap by thoroughly assessing microalgal bioremediation in urban wastewater. Specific objectives are: (i) to evaluate the growth of a native microalgae-bacteria consortium from UWW in secondary-treated effluent collected from a Portuguese urban wastewater treatment plant (UWWTP); (ii) to assess the microalgae capacity to remove conventional pollutants from the effluent to meet the EU legislation (Council Directive 1991/271/EEC) [21], also determining the process kinetics; (iii) to analyse the effect of microalgae growth on coliform (*E. coli* and other coliforms except *E. coli*) removal; and (iv) to evaluate the impact of microalgae cultivation on the reduction of *intI*1 and antibiotic resistance genes (*sul*1 and *bla*_TEM_) abundance and prevalence during effluent treatment and treated water storage.

## 2. Materials and Methods

### 2.1. Urban Wastewater

The effluent was sourced from an UWWTP in the northern region of Portugal, corresponding to a population of 65,000 inhabitants with a maximum daily average flow of 10,000 m^3^ of UWW. The wastewater treatment process at this UWWTP involves: (i) a preliminary treatment, employing automatic grading and screening in a rotary drum for the removal of solids and coarse elements, followed by sand removal through compressed air injection; (ii) a primary treatment, where sludge is separated from grease and oils in sedimentation tanks; and (iii) a secondary or biological treatment, utilising an activated sludge system comprising four sequencing batch reactors to reduce dissolved and suspended organic matter. Effluents were collected for post-secondary treatment, promptly transported to the laboratory, and subjected to further processing.

### 2.2. Microalgae Consortium

In this study, a native microalgae-bacteria consortium was used. For that, microalgal growth was induced from a UWW sample (40 L) primarily collected from the secondary treatment of the same UWWTP by exposing UWW to a 24:0 light cycle light. UWW was kept in a 120 L photobioreactor until the microalgal density was satisfactory for use (approximately four weeks). To maintain nutrients at desired levels, a modified OECD (Organisation for Economic Cooperation and Development) test medium (see composition in Table S1—Supplementary Material) was added weekly to the culture.

### 2.3. Experimental Setup

UWW treatment with the MBS was carried out in triplicate in 2-L aerated photobioreactors (PBRs), with a working volume of 2 L, and operated in batch mode (see Figure S1). To validate the microbiological results, the following control reactors were implemented: (i) a positive control (C+), consisting of autoclaved UWW inoculated with MBS; (ii) a negative control (C−), consisting of non-inoculated raw UWW; and (iii) a dark negative control (DC−), consisting of non-inoculated raw UWW kept under dark conditions throughout the experiment. The cultures were monitored daily regarding temperature and pH (Consort’s C6010 electrochemical analyser, Brussels, Belgium). The experiments were performed with no pH control, aiming to study microalgal growth and pollutants’ removal under high pH (above 8.5) imposed by microalgal photosynthesis, creating hostile conditions for pathogens (namely faecal coliforms) [7].

PBRs (MBS and C+) were initially inoculated with a microalgal cell concentration (MCC) of 4 × 10^6^ cells mL^−1^. These reactors operated continuously for 6 days under white light-emitting diodes (4000 K) at a light intensity of 400 µmol m^−2^ s^−1^, with a photoperiod of 24 h of light and no dark cycle. Atmospheric air was supplied at a rate of 0.3 VVM (volume of air per volume of culture per minute) using a Sicce Airlight 3300 (Pozzoleone, Italy). The ambient room temperature was 18 ± 3 °C, and any potential evaporation effects were considered negligible.

Furthermore, the feasibility of reusing the treated water was explored. To achieve this, at the end of the experiment, microalgal cells were harvested through centrifugation at 1431× *g* using an Eppendorf 5804 R centrifuge (Hamburg, Germany) for 10 min. Subsequently, the supernatant (treated water) was carefully stored in sealed flasks for 8 days in dark conditions at room temperature.

### 2.4. Microalgal Growth Kinetics

The microalgal biomass were monitored daily. Microscopic determination using a Neubauer chamber (BlauBrand, Steinheim, Germany) with a LEICA DMLB microscope (type 020-519.502, Wetzlar, Germany) was employed to quantify the MCC in each reactor. To establish a calibration curve relating MCC to biomass concentration in terms of dry weight (g_DW_ L^−1^), samples were collected daily for subsequent filtration through pre-dried cellulose acetate filters (0.45 µm; Sartorius, Gottingen, Germany), followed by drying for 24 h at 105 °C. After cooling to room temperature in a desiccator, the filters were reweighed to calculate biomass concentration in terms of dry weight. Additionally, autochthonous microalgal classes were identified based on their morphology through microscopic observation at 400× magnification.

The specific growth rate (*μ*, d^−1^) during the exponential growth phase was determined by fitting the time-series MCC data to the modified Gompertz model [22], as represented in Equation (1). In this equation, A represents the MCC at the stationary growth phase (cells mL^−1^), and λ is the lag time (d). To assess the model’s performance, the coefficient of determination (R^2^) and the root mean squared error (RMSE) were calculated according to Equations (2) and (3), respectively. In these equations, $y_{i}$ are the experimental values, ${\hat{y}}_{i}$ are the predicted model values, $\bar{y}$ is the average of the experimental values, and n represents the number of data points.

(1) $$\ln\left(\frac{MCC\left(t\right)}{MCC(0)}\right)=A\cdot exp\left\{-\exp\left[\frac{\mu \cdot e}{A}\left(\lambda -t\right)+1\right]\right\}$$

(2) $$R^{2}=1-\frac{\sum_{i=1}^{n}{\left(y_{i}-{\hat{y}}_{i}\right)}^{2}}{\sum_{i=1}^{n}{\left(y_{i}-\bar{y}\right)}^{2}}$$

(3) $$RMSE=\sqrt{\frac{\sum_{i=1}^{n}{\left(y_{i}-{\hat{y}}_{i}\right)}^{2}}{n}}$$

Biomass productivities, $P_{X}$, were computed using Equation (4) for each pair of consecutive data points. In this equation, $X_{i}$ represents the biomass concentration at the time $t_{i}$ and $X_{i+1}$ represents the biomass concentration at the time $t_{i+1}$. Maximum biomass productivity ($P_{X,max}$) corresponds to the highest values calculated through this equation. To determine the average biomass productivity ($P_{X,avg}$), Equation (5) was employed. $X_{f}$ and $X_{0}$ correspond to the biomass concentration at times $t_{f}$ and $t_{0}$, which correspond to the end and the beginning of the experiment, respectively.

(4) $$P_{X}=\frac{X_{i+1}-X_{i}}{t_{i+1}-t_{i}}$$

(5) $$P_{X,avg}=\frac{X_{f}-X_{0}}{t_{f}-t_{0}}$$

### 2.5. Nutrient Removal Efficiency and Kinetics

Nutrient removal from UWW was monitored daily, with additional assessments conducted for the treated water following designated storage periods on days 3 and 8 (St3 and St8, respectively). To achieve this, 30 mL samples were extracted from each PBR and subjected to centrifugation at 1431× *g* using an Eppendorf 5804 R centrifuge (Hamburg, Germany) for 10 min, and the resulting supernatants were promptly frozen at −20 °C for subsequent analysis.

The supernatants were subjected to characterisation (duplicate analyses for MBS and triplicate for C+, C−, and DC−) encompassing: (i) Chemical Oxygen Demand (COD) measured in mg L^−1^, according to the 5220-D procedure outlined in the Standard Methods for the Examination of Water and Wastewater [23]; (ii) Nitrate-nitrogen (NO_3_^-^-N) in mg L^−1^, employing the United States Environmental Protection Agency (USEPA)-approved Brucine Colourimetric Method [24]; (iii) Ammonium-nitrogen (NH_4_^+^-N) concentration measured in mg L^−1^, utilising the Spectroquant Ammonium Kit Test (Merck, Darmstadt, Germany) and a Spectroquant Prove 300 spectrophotometer (Merck, Darmstadt, Germany); (iv) Phosphate-phosphorus (PO_4_^3−^-P) levels measured in mg L^−1^, following the ascorbic acid colourimetric method as described by Lee and Lei [25]; and (v) HA concentration, indirectly determined through UV spectroscopy at 254 nm using a PG Instruments Ltd., T80 UV/VIS Spectrometer. The concentrations of COD, NO_3_^−^-N, PO_4_^3−^-P, and HA were determined by the calibration curves presented in Table S2. The input concentrations of COD, NH_4_^+^-N, and PO_4_^3−^-P in the UWW were 74.9 mg L^−1^, 65.0 mg L^−1,^ and 1.3 mg L^−1^, respectively.

The calculation of nitrogen and phosphorus mass removal (MR) and average removal rate (RR) were based on Equations (6) and (7), with $S_{0}$ and $S_{f}$ representing the nutrient concentrations in mg L^−1^ at days 0 (t_0_) and 6 (t_f_), respectively. Nutrient removal efficiencies (RE) were determined using Equation (8).

(6) $$MR=S_{0}-S_{f}$$

(7) $$RR=\frac{MR}{t_{f}-t_{0}}$$

(8) $$RE\left(\%\right)=\frac{S_{0}-S_{f}}{S_{0}}\times 100$$

The experimental data tracking nitrogen and phosphorus removal over time was subjected to fitting using the modified Gompertz model [22], as described in Equation (9). In this equation, *k* represents the nutrient uptake rate (d^−1^), and *λ* is the lag time (d). These kinetic parameters were determined. To assess the model’s performance, the calculations of the $R^{2}$ and the RMSE were again performed, following Equations (2) and (3), respectively.

(9) $$S\left(t\right)=S_{0}+(S_{f}-S_{0})\times exp\left\{-\exp\left[k\cdot \left(\lambda -t\right)+1\right]\right\}$$

The biomass yield based on nutrient consumption ($Y_{X/S}$, g_DW_ g_nutrient_^−1^) was calculated according to Equation (10).

(10) $$Y_{X/S}=\frac{X_{f}-X_{0}}{S_{0}-S_{f}}$$

### 2.6. Culturable Microorganisms’ Determination

Throughout the study, the total heterotrophic and coliform bacteria populations were monitored, including *E. coli* and other coliforms except *E. coli*, within each PBR. Duplicate 1 mL samples were collected on days 0, 3, 6, and after 3 days of storage, serving as the basis for enumerating microorganisms. The enumeration process involved the application of the membrane filtration method, wherein 1 mL of each serially 10-fold diluted sample was filtered through cellulose nitrate membrane filters (0.22 µm porosity; Whatman, Maidstone, UK). Subsequently, the filtered membranes were incubated on Plate Count Agar (Merck, New York, NY, USA) at 30 °C for 48 h to quantify total heterotrophs.

For the specific enumeration of *E. coli* and other coliforms, Chromogenic Coliform Agar was utilised, following the established procedure outlined by Lange et al. [26]. Incubation of these plates at 36 °C for 24 h revealed *E. coli* colonies in a distinctive blue hue, while other coliforms manifested as pink colonies.

### 2.7. DNA Extraction

In addition to bacterial enumeration, DNA extraction was performed from 50 mL samples taken from each PBR on days 0, 3, 6, and after 3 and 8 days of the storage period. These samples were filtered using polycarbonate membranes (0.22 µm porosity; Whatman, Maidstone, UK), and subsequent extraction of total deoxyribonucleic acid (DNA) was performed utilising the Power Soil^®^ DNA Isolation commercial kit (MO BIO Laboratories, Inc., Carlsbad, CA, USA) following the manufacturer’s protocol. The concentration of the extracted total DNA was quantified using the Qubit 3.0 Fluorometer (Invitrogen, Waltham, MA, USA) equipped with the Qubit^®^ dsDNA HS assay kit (Invitrogen, USA). The obtained DNA samples were then stored at −20 °C until further analysis.

### 2.8. Quantification of 16S rRNA, intI1, and Antibiotic Resistance Genes

Quantitative polymerase chain reaction (qPCR; StepOne^TM^ Real-Time PCR System, Life Technologies, Carlsbad, CA, USA) was performed in duplicate for each DNA extract. This was completed to quantify the class 1 integrons integrase gene (*intI*1), the selected antibiotic resistance genes (ARGs) *sul*1 and *bla*_TEM_, and the 16S rRNA gene as a measure of total bacterial abundance using the conditions presented in Table 1. Quantification and determination of the abundance of each gene (GA), ARG prevalence, and percentage reduction of gene prevalence (RGP) were performed. Quantification was performed utilising the standard curve method as outlined in the StepOne™ Real-Time PCR System manual (Life Technologies, Carlsbad, CA, USA) [27]. To address potential qPCR inhibition, target genes were assessed in a serially diluted sample following the methodology proposed by Bustin et al. [28]. GA was expressed as the number of gene copies per 100 mL. The initial concentration of bacteria in MBS PBRs, indicated by 16S rRNA abundance, was (1.4 ± 0.3) × 10^9^ gene copies per 100 mL^−1^. Therefore, the initial microalgae-to-bacteria ratio was approximately 1:3 (0.32 ± 0.05).

To evaluate *intI*1 and ARG prevalence, GA was normalised with the 16S rRNA abundance value for the corresponding sample (gene copy number per 100 mL/16S rRNA copy number per 100 mL). Additionally, the percentage reduction of gene prevalence (RGP) was calculated using Equation (11), where ${GRA}_{0}$ and ${GRA}_{f}$ represent the gene relative abundance at the beginning and end of the experiment, respectively.

(11) $$RGP\;(\%)=\frac{{GRA}_{0}-{GRA}_{f}}{{GRA}_{0}}\times 100$$

The average decrease rate of gene prevalence (DRGP, gene copy number/16S rRNA copy number/d) was also calculated during the studied period.

### 2.9. Statistical Analysis

The analysis of the experimental data was conducted. A two-way ANOVA with Tukey’s multi-comparison test was used to compare the studied variables (MCC, pH, chemical oxygen demand—COD, HA, NH_4_^+^-N, PO_4_^3−^-P) over time and between the PBRs. A One-way ANOVA was used to compare growth parameters (MCCmax, PX,avg, and PX,max) and nutrient uptake parameters (MR and RE) between PBRs and DRGP. A Pearson correlation analysis was performed among all the studied variables within each PBR. All statistical analyses were conducted at a significance level of 0.05.

Modified Gompertz models and their performance indexes (R^2^ and RMSE) were determined using Microsoft Excel (2301, Microsoft, Washington, DC, USA).

## 3. Results and Discussion

### 3.1. Microalgal Growth

**Figure 1:**
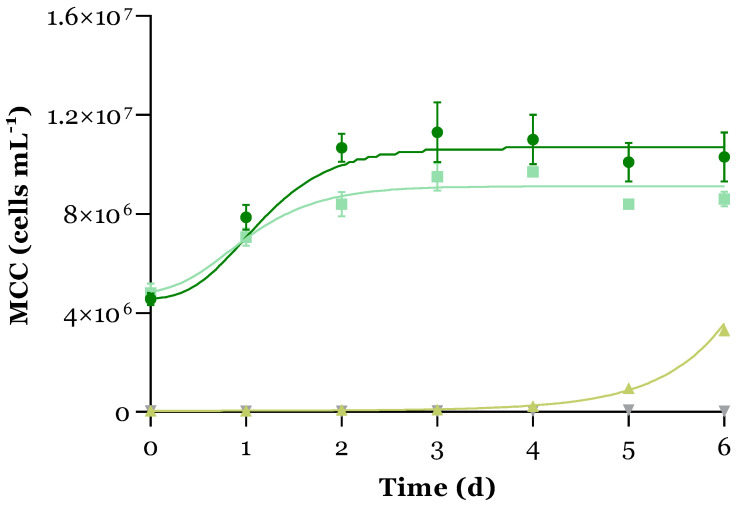
Microalgal cell concentration (MCC, average ± standard deviation, n = 3) over time (d) for each PBR: 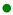 MBS; 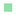 C+; 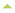 C−; and 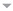 DC−. The filled line represents the model fit of the modified Gompertz model to the experimental data.

Figure 1 shows the microalgal growth profiles across the UWW treatment, while Table 2 provides the calculated growth parameters. Microalgae grew successfully in the MBS PBR, which exhibited the highest specific productivity values (P_X,max_ and P_X,avg_) and a specific growth rate of (0.651 ± 0.155) d^−1^. Silva et al. [33] obtained similar specific growth rates (0.60–0.68 d^−1^) when culturing *C. vulgaris* in comparable conditions. A brief lag phase was obtained with the modified Gompertz model fitting (0.324 ± 0.176 d and 0.470 ± 0.131 d, for MBS and C+, respectively), proving the adaptability of this microalgae to the UWW. However, the stationary phase was attained approximately after day 3, probably due to the extreme alkaline pH (>11) conditions in the culture medium in these PBRs that may limit the availability of some important nutrients for microalgal growth. The diversity of microalgal species present in the MBS at the beginning of the experiments was characterised (see Figure S2). The microalgal species belonged to the microalgal classes *Cyanophyceae*, *Chlorophyceae*, and *Bacillariophyceae*.

Autochthonous microalgae started to increase exponentially in the C− PBR after day 3 (λ = 2.996 ± 0.860 d), attaining similar productivity values to the C+ PBR and a 3-fold higher specific growth rate than the C+ and MBS PBRs (1.442 ± 0.451 d^−1^). This may be explained by the combination of biotic and abiotic factors, mainly light intensity. This is corroborated by the fact that no microalgal growth was observed in the DC− PBR, in which the only difference from the C− PBR was the absence of light.

Regarding biomass productivity, MBS attained (1.9 ± 0.6) × 10^−4^ g_DW_ mL^−1^ d^−1^. This value is in agreement with the ones obtained by Silva et al. [33] (1.06 × 10^−4^ g_DW_ mL^−1^ d^−1^) for *C. vulgaris* growing in analogous conditions. A calibration curve between the time course data of MCC and biomass concentration in the exponential phase was implemented (see Table S2). The average dry weight of the microalgal cells in the consortium is given by the inverse of the slope of this calibration curve: (3.9 ± 0.5) × 10^−11^ g_DW_ cell^−1^. Hu [34] reported an average *C. vulgaris* dry weight at the exponential phase of (2.24 ± 0.16) × 10^−11^ g_DW_ cell^−1^ (n = 12), which is in the same order of magnitude as the one obtained in the present study.

The pH variation over time was assessed for all PBRs (see Figure S3). The initial pH values were consistent between the inoculated PBRs and the negative control PBRs. This observation underscores that the introduction of the inoculum had no apparent impact on the pH of the wastewater. As expected, the C− and DC− PBRs presented analogous pH variation until day 3, given that microalgae did not develop in these PBRs. Their pH stabilised around 8.5, slightly above the initial pH (7.5 ± 0.1). The pH of the MBS increased significantly in the first 2 days (*p* < 0.05), stabilising at a pH of around 11.4. The same happened in the C− PBR after native microalgae concentration started to increase exponentially after day 3. This is common in microalgal systems due to the HCO_3_^-^ conversion to CO_2_ during photosynthesis, releasing OH^-^ ions [7]. After the microalgal UWW treatment, the pH of the treated water decreased to neutral pH (after 8 days of storage under dark conditions), allowing its reuse or discharge in natural environments without extra chemical pH adjustments.

### 3.2. Conventional Pollutant Removal

**Figure 2:**
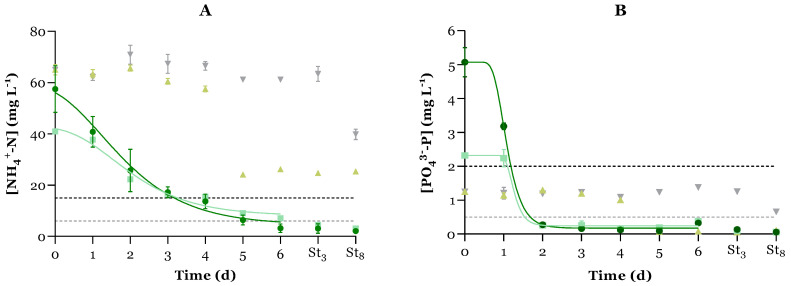
Variation of ammonium-nitrogen ((**A**) NH_4_^+^-N, mg L^−1^) and phosphate-phosphorus ((**B**) PO_4_^3−^-P, mg L^−1^) concentrations (average ± standard deviation, n = 3) over time (d) for each PBR: 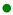 MBS; 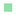 C+; 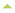
C−; and 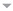 DC−. The filled lines represent the model fit of the modified Gompertz model to the experimental data. The dashed lines correspond to the EU legislation limits (directive 1991/271/EEC—black; upgrade for 2040—grey). St_3:_ day 3 of storage; St_8_: day 8 of storage.

One of the primary advantages of employing microalgae-based treatments is their capacity to efficiently remove conventional pollutants, such as nitrogen and phosphorus. According to Council Directive 1991/271/EEC, which pertains to UWWTPs serving fewer than 100,000 population equivalents, the permissible discharge limits for total nitrogen and phosphorus in effluents are 15 mg L^−1^ and 2 mg L^−1^, respectively [21]. Furthermore, the European Commission has proposed an update to this directive, which mandates stricter limits, requiring levels to be reduced below 6 mg L^−1^ for total nitrogen (TN) and 0.5 mg L^−1^ for total phosphorus (TP) by 2040 [35]. Hence, the potential of the microalgal-bacterial consortium to reduce the concentrations of these conventional pollutants to values below the legal limits was assessed. The temporal evolution of ammonium-nitrogen and phosphate-phosphorus concentrations is shown in Figure 2. Subsequently, the initial concentration, removal parameters, and efficiency after 6 days were calculated (see Table 3). Additionally, the experimental data was fitted to the modified Gompertz model (Equation (9)), and the resulting kinetic parameter k (d^−1^) is also presented in Table 3. The same analysis is not presented for NO_3_^-^-N since its initial concentration was already below the detection limit (0.26 mg L^−1^), and no NO_3_^-^-N was produced during the 6 days of the experiment in any of the PBRs.

The results showed that the initial level of NH_4_^+^-N in the effluent was significantly higher than the limits imposed by the current legislation (15 mg L^−1^), whereas the concentration of phosphorus was below the legal limit (2 mg L^−1^), indicating that this UWWTP might need to apply tertiary treatment to its effluents in the future. The profiles for NH_4_^+^-N and PO_4_^3−^-P removal over time in C− (Figure 2A and Figure 2B, respectively) are coherent with microalgal growth since, from day 3, autochthonous microalgae started to increase exponentially, actively consuming these nutrients. Indeed, a strong negative Pearson’s correlation was established between the levels of these nutrients and MCC over time (*r* < −0.9, *p* < 0.05—see Figure S4). Overall, the MBS could efficiently remove NH_4_^+^-N and PO_4_^3−^-P (*r* < −0.9, *p* < 0.05), reaching values below the current EU legislation limits within the cultivation time. The N:P mass ratio (11.42 ± 2.49) in the MBS was within the optimal range (5–30) for microalgal growth [7].

Regarding the NH_4_^+^-N removal, MBS showed the best results in terms of mass removal and removal efficiency (see Table 3), attaining REs above 92%. Although MR and RE were lower in the C+ PBR in relation to the MBS (due to the lower initial concentration in C+), the NH_4_^+^-N uptake rate was similar between the two PBRs (*p* > 0.05). The uptake rate achieved by the MBS is in line with the ones obtained by Salgado et al. [9] during *C. vulgaris* cultivation with controlled pH (around 7) in synthetic wastewater, with N:P ratios of 9–14 (0.3 ± 0.2 d^−1^ to 1.1 ± 0.2 d^−1^). The biomass yield due to NH_4_^+^-N consumption (Y_X/S_) was similar between experiments, corresponding to a percentage of nitrogen in biomass of about 13%. Considering that this parameter is typically 7–12%, this indicates that ammonia stripping was not significant [36].

PO_4_^3−^-P removal using the MBS was efficient, revealing a high RE and uptake rate: 94 ± 1% and 3.829 ± 1.330 d^−1^. The high pH can explain this since PO_4_^3−^-P can be indirectly removed by precipitation through the complexation with Ca, Mg, and Fe at pH > 8 [7]. The kinetic constant was significantly higher than those obtained at neutral pH by Salgado et al. [9] (0.4 ± 0.1 d^−1^ to 0.6 ± 0.1 d^−1^), suggesting that PO_4_^3−^-P precipitation played an important role in phosphorus removal. This is corroborated by the Y_X/S_ value obtained (85.94 ± 28.61 g_DW_ g_P_^−1^), corresponding to a phosphorus content in microalgae of ca. 1%. According to Powell et al. [37], microalgae typically contain a maximum phosphorus content of 1% without luxury uptake. Therefore, luxury uptake or phosphorus precipitation improved PO_4_^3−^-P removal in the experiment.

After microalgal treatment, the treated water appears stable regarding NH_4_^+^-N and PO_4_^3−^-P concentrations during the storage period (Figure 2). The more extended storage period (8 days, St_8_) led to a significant decrease in NH_4_^+^-N concentration in the DC− PBR, which can be directly correlated with nitrifying bacteria activity since higher levels of NO_3_^-^-N were registered in this PBR at St_8_ (5.66 ± 0.36 mg L^−1^) than in the rest of the experiment (values lower than the limit of detection).

**Figure 3:**
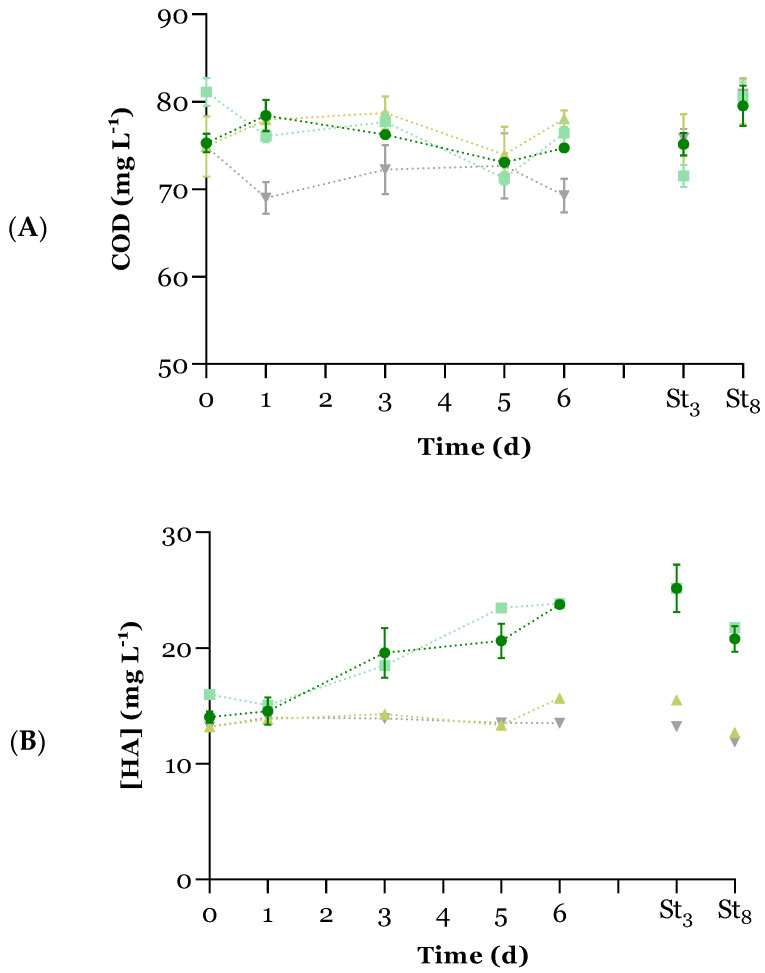
Variation of (**A**) chemical oxygen demand (COD, mg L^−1^) and (**B**) humic acids (HA, mg L^−1^) concentration (average ± standard deviation, n = 3) over time (d) for each PBR: 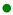
MBS; 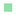 C+; 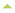 C−; and 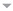 DC−. St_3_: day 3 of storage; St_8_: day 8 of storage.

Regarding carbon, the variation of COD concentrations over time in all PBRs was monitored (see Figure 3A). The initial UWW COD concentration was below the limit established by Council Directive 1991/271/EEC (125 mg O_2_ L^−1^) [21]. In the C+ PBR, the UWW sterilisation caused a slight increase in the initial COD concentration, which can be related to organic matter degradation or concentration effects due to water evaporation during autoclaving. Overall, microalgal UWW treatment did not affect the COD levels within the cultivation time since no significant differences were registered between days 0 and 6 for MBS (*p* > 0.05). This proves that a microalgae-bacteria system can be implemented in a UWWTP as a tertiary treatment without further increasing the organic matter content. A COD decrease was observed from day 1 to day 5 (in MBS and C+, *p* < 0.05), meaning that either microalgae went through a mixotrophic metabolism phase or the consumption by the bacteria from the inoculum and/or UWW was more substantial. On the other hand, in the negative control PBRs (C− and DC−), COD concentration oscillated over time. While a decrease in COD levels may be attributed to heterotrophic bacterial consumption of organic matter, an increase in COD values may be related to bacterial excretion of metabolic products, such as EPS, of which humic acids are a component. In fact, previous studies have shown a correlation between COD and HA concentrations in MBS (*r* between 0.7 and 0.8) [8]. Hence, the variation in the content of humic acids over time was evaluated for all PBRs (see Figure 3B). However, no correlation was found between COD and HA levels for any PBRs (*r* < 0.6).

Nevertheless, in MBS PBRs, HA concentration increased during the 6 days of cultivation (*p* < 0.05). Microalgal species are known to naturally secrete EPS, which contain humic acids, especially when adapting to certain stress factors such as unfavourable light, temperature, and pH conditions, low nutrient availability, or the presence of toxic substances [38]. Thus, the extreme alkaline pH observed (>11 from day 2) combined with nitrogen and phosphorus scarcity from day 3 may have induced HA production by microalgae, increasing from 14.3 ± 0.9 mg L^−1^ in the exponential growth phase (until day 3) to 22.2 ± 2.0 mg L^−1^ in the stationary phase. These observations are in agreement with Raungsomboon et al. [39], who reported a higher amount of EPS secreted by the cyanobacteria *Gloeocapsa gelatinosa* during stationary (24.1 ± 1.0 mg L^−1^, 20 d) and death (33.0 ± 1.0 mg L^−1^, 30 d) phases than in the log phase (15.0 ± 1.0 mg L^−1^, 10 d).

### 3.3. Cultivable Bacterial Abundance

**Figure 4:**
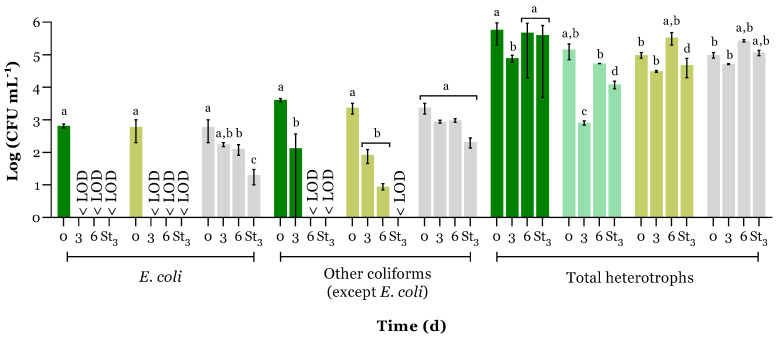
Bacterial quantification, namely in terms of *E. coli*, other coliforms except *E. coli*, and total heterotrophs over the experiment time (d) and after 3 days of treated water storage (St_3_), for each PBR: 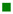 MBS; 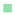 C+; 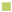 C−; and 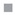 DC− Results are expressed in log (CFU mL^−1^) as the average ± standard deviation (n = 3). Bars from the same group of bacteria with a different superscript letter are statistically different (two-way ANOVA followed by a Tukey post hoc test, *p* < 0.05).

Bacterial abundance was assessed for all PBRs during the wastewater treatment (6 days) and after a storage period of 3 days in the treated water. Microbial abundance was determined with a focus on *E. coli*, other coliforms (excluding *E. coli*), and total heterotrophic bacteria (see Figure 4). As expected, in the C+ PBR, the autoclave sterilisation process led to the complete absence of coliforms. Hence, in this type of control PBR, only total heterotrophs originating from the microalgae inoculum were detected at t0 (Figure 4).

As discussed above, the microalgae growth in the MBS PBR was concomitant with the pH increase. This rise in pH may have had a significant impact on reducing the abundance of *E. coli* and other coliforms (excluding *E. coli*) to levels below the limit of quantification (LOQ, 5 CFU mL^−1^) after 3 and 6 days, respectively. Furthermore, the total coliform abundance, including *E. coli*, exhibited a negative correlation with microalgal growth, pH elevation, and HA production (*r* < −0.9 and *p* > 0.05). Additionally, the development of native microalgae in the C− PBR led to a reduction in the abundance of *E. coli* to values lower than the limit of quantification and to a significant decrease in other coliforms within 6 days, although to a lesser extent than in the MBS PBR. This suggests that *E. coli* is less resistant to environmental stressors than other coliforms, as Halkman and Halkman (2014) described. Several studies also reported the faster inactivation of *E. coli* in relation to other coliforms when the pH is above 8.5 and also due to the photo-oxidative damage caused by exogenous photosensitisers on cell membranes [16,40]. According to Cheng et al. [41], the wide disparity in pH values between microalgae-cultivated PBRs and the DC− PBR may be the main contributor to this distinct performance.

The MBS presented small variations in total heterotroph abundance over time. This suggests that the microalgae-bacteria consortium was well established, allowing microalgae and bacteria to proliferate symbiotically, which improves wastewater bioremediation [42]. While microalgae produce O_2_ and organic matter, aerobic bacteria use O_2_ for respiration, consume the organic compounds, and provide CO_2_ and vitamins to enhance microalgae [8].

Ribeirinho-Soares et al. [43] reported that the regrowth of microorganisms during the storage period after some disinfection processes, such as ozonation, is a consistent occurrence. However, in the present study, the 3-day storage of the MBS PBR treated water did not induce bacterial regrowth, allowing its safe reutilisation, for instance, in agricultural practices [44].

### 3.4. Abundance of Antibiotic Resistance Genes

**Figure 5:**
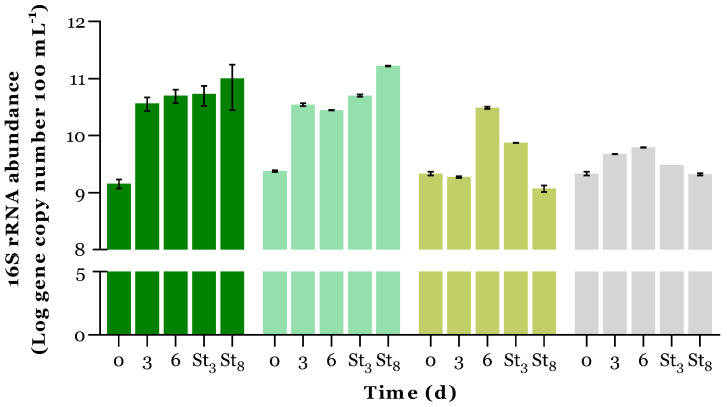
Abundance of 16S rRNA gene over time (d) in each PBR: 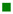 MBS; 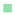 C+; 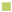 C−; and 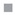 DC−. Results are expressed in log (gene copy number 100 mL^−1^) as the average ± standard deviation (n = 2). St_3:_ day 3 of storage; St_8_: day 8 of storage.

**Figure 6:**
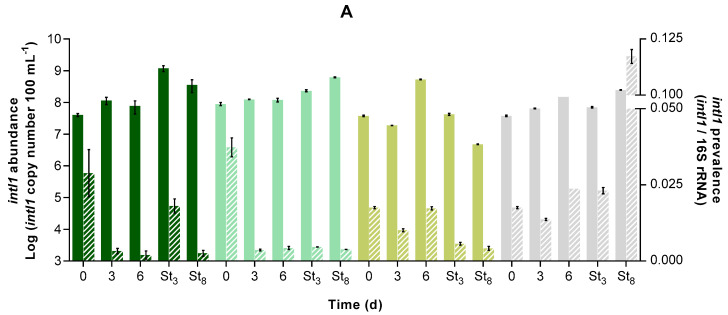

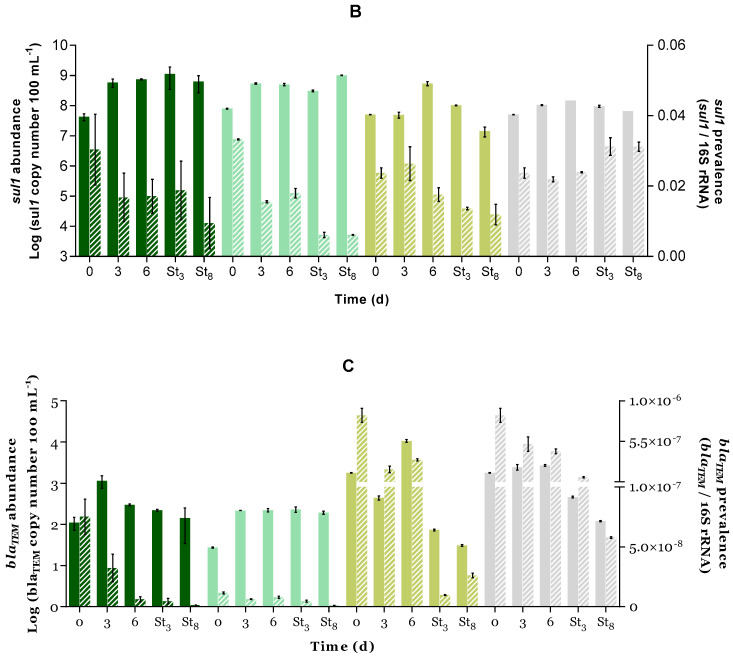
Abundance (left y-axis, full coloured bars) and prevalence (right y-axis, stripped bars) of *intI*1 (**A**), *sul*1 (**B**), and *bla_TEM_* (**C**) genes over time (d) in each PBR: 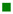. MBS; 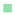 C+; 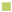 C−; and 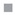 DC−. Results are expressed as average ± standard deviation (n = 2). St_3_: day 3 of storage; St_8_: day 8 of storage.

The total bacterial abundance (cultivable and non-cultivable) was estimated by quantifying the housekeeping gene 16S rRNA for all the PBRs (Figure 5). The abundance of *intl*1, encoding for class 1 integron integrase and a proxy for anthropogenic contamination [45], and the ARGs *sul*1 and *bla*_TEM,_ respectively related to sulfonamides and beta-lactam resistance, were also quantified (Figure 6). ARGs and *intI*1 prevalence over time were also determined through the normalisation of GA with the respective 16S rRNA prevalence values (stripped bars in Figure 6). Table 4 presents the initial GA values and the percentage reduction in gene prevalence (from days 0 to 6).

As expected, the abundance of total bacteria was around 2 log higher than when quantified by cultivable methods (Figure 5 vs. Figure 4). At day 6, this ratio was higher than 3 log, indicating that the overall cultivability of total bacteria was reduced. Overall, an increase in 16S rRNA generally occurred over the cultivation time. This increase was more prominent in the PBRs with microalgal growth (MBS, C+, and C−), and indeed, a strong positive Pearson’s correlation was established between 16S rRNA abundance and MCC over time (*r* > 0.9, *p* < 0.05) for C+ and C− PBRs, reinforcing the importance of symbiotic interactions between microalgae and bacteria for biomass production.

Regarding *intI*1 and *sul*1 initial abundance (A_0_, Table 4), similar values were found between the MBS, C−, and DC− PBRs for each gene. On the other hand, the initial *bla_TEM_* abundance was higher in the negative control PBRs (raw wastewater) than in the inoculated PBRs (MBS and C+). Hence, the dilution effect caused by microalgae inoculation may have contributed to the differences observed. Furthermore, *bla_TEM_* was the least abundant gene (4 to 5 log fewer gene copies per mL than *intI*1 and *sul*1). Cacace et al. [46] also reported a higher abundance and dissemination of *intI*1 and *sul*1 across European countries compared to *bla*_TEM_ (labelled as an intermediately abundant gene). The results showed that *intI*1 abundance was maintained during the cultivation period in the inoculated PBRs or even increased over time (6 days) in the non-inoculated PBRs (Figure 6). In contrast, the abundance of *sul*1 and *bla_TEM_* increased by around 1 log in the first 3 days in the inoculated PBRs, whereas in the non-inoculated PBRs, the increase was less significant over the 6 days of incubation.

To evaluate the effect of stress factors on gene abundance, the values should be normalised by the content of 16S rRNA [47], defined as gene prevalence. The prevalence of *intI*1, *sul*1, and *bla*_TEM_ genes was reduced by over 93%, 42%, and 91.7%, respectively (corresponding to an average DRGP of 4.8 × 10^−3^, 2.4 × 10^−3^, and 1.2 × 10^−8^ gene copy number/16S rRNA copy number/d—see Table S3), during the 6 days in the MBS PBR. In the C+ PBR, the achieved decrease kinetics did not present statistically different parameters compared to MBS (*p* < 0.05). In the C− PBR, the prevalence of the ARGs decreased, but to a lesser extent than in the MBS PBR. However, in the DC− PBR (no microalgae growth), the prevalence of *intI*1 increased, *sul*1 was approximately constant, and *bla_TEM_* decreased. The prevalence decrease indicates that the increase in total bacterial abundance was greater than the increase in *intI*1 and ARGs over time. These results suggest that the presence of microalgae favoured the growth of bacteria not carrying *intI*1 and the antibiotic resistance genes analysed in this work.

Ovis-Sanchez et al. [48] attributed the significant removals of ARGs and *intl*1 to reductions in ESKAPEE bacteria (*Enterococcus faecium*, *Staphylococcus aureus*, *Klebsiella pneumoniae*, *Acinetobacter baumannii*, *Pseudomonas aeruginosa*, *Enterobacter* spp., and *E. coli*) since they are often reservoirs of ARGs. In fact, a high Pearson’s correlation coefficient (*r* > 0.9) was determined between the prevalence over time of total coliforms (*E. coli* and other coliforms) and *sul*1 (*p* < 0.05) and *bla_TEM_*, in the MBS, as well as between the prevalence over time of *bla_TEM_* and total coliforms in C− and DC− PBRs. However, in the present study, the abundance of the targeted genes was maintained or even increased over time, suggesting that the carriers of the analysed genes were non-coliform bacteria. Hence, further studies on the composition of the bacterial community associated with the microalgae in MBS PBRs are necessary.

The treated wastewater was also tested for its stability in terms of 16S rRNA, *intI*1, and ARG content after 3 and 8 days of storage under dark conditions to test the possibility of water reutilisation. In microalgae-treated water, the abundance of the studied genes followed the pattern described before, stabilising or increasing their abundance during the storage period. On the contrary, in C− PBR, a decrease in both gene abundance (2.0, 1.6, and 2.5 log for *intI*1, *sul*1, and *bla*_TEM_, respectively) and prevalence was recorded from the last day of treatment to the last day of storage, suggesting that the native microalgae consortium grown in this PBR was more efficient at ARG removal. Similar results were found in a previous study [8], in which the native microalgae growth in the negative control PBR caused a reduction of *intl*1 (1 log), *sul*1 (0.5 log), and *bla*_TEM_ (2.5 log) abundance and prevalence after a storage period of 5 days, relatively to the last day of treatment (9 days). The authors attributed this to bacteria adsorption to microalgal cells and further co-precipitation. However, since the same did not occur in MBS and C+ PBRs in the present study, this phenomenon might be related to the higher pH values recorded during storage of C− (Figure S3), imposing a different bacterial community structure and composition. Nevertheless, except for the DC− PBR, the prevalence of all the analysed genes was lower during the storage period than at the beginning of the experiments (Figure 6). Hence, storage for 8 days led to a decrease in *intI*1, *sul*1, and *bla*_TEM_ prevalence in at least 90%, 71%, and 98%, respectively, in the microalgae-treated wastewater (Table 5). Conversely, DC− PBR triggered the increase of *intI*1 and *sul*1 prevalence (RGP < 0), attaining higher relative abundances at day 8 of storage than the other PBRs. Hence, although ARG-carrying bacteria were in higher abundance than in the initial wastewater, MBS seems to have stimulated the growth of non-ARG-carrying bacteria.

## 4. Conclusions

This study explores urban wastewater treatment using a native microalgae-bacteria consortium. Compliance with EU pollutant limits occurred in 2 to 4 days, with NH_4_^+^-N and PO_4_^3−^-P removal exceeding 94% and 93%, respectively. Elevated pH and light exposure may have been the main factors that eradicated *E. coli* and coliforms within 3 and 6 days. Antibiotic resistance gene prevalence decreased substantially—over 92%, 42%, and 91% for *intI*1, *sul*1, and *bla*_TEM_, respectively, within 6 days. MBS-treated water, stored in the dark for 3 days, showed no bacterial regrowth, and further reductions in *sul*1 and *bla*_TEM_ prevalence occurred after 8 days (achieving a total reduction of approximately 72% and 98%, respectively).

## Figures and Tables

**Table 1:** Conditions for qPCR assays.

Gene	Primers	qPCR Standard	Efficiency (%)	Conditions	Master Mix	Ref.
16S rRNA (145 bp)	q_1114F (CGGCAACGAGCGCAACCC) q_1275R (CCATTGTAGCACGTGTGTAGCC)	*Escherichia coli* ATCC25922	100	95 °C for 10 min (1 cycle); 95 °C for 15 s, 55 °C for 20 s, and 72 °C for 10 s (35 cycles)	KAPA SYBR^®^ FAST, ABI Prism^®^	[29]
*intl*1 (196 bp)	q_intILC5_fw (GATCGGTCGAATGCGTGT) q_intILC1_rv (GCCTTGATGTTACCCGAGAG)	pNORM (digested)	94	95 °C for 10 min (1 cycle); 95 °C for 15 s, and 60 °C for 1 min (40 cycles)	PowerSYBR^®^ Green PCR Master Mix	[30]
*sul*1 (162 bp)	q_sulI_FW (CGCACCGGAAACATCGCTGCAC) q_sulI_RV (TGAAGTTCCGCCGCAAGGCTCG)	pNORM (digested)	94	95 °C for 5 min (1 cycle); 95 °C for 10 s, and 60 °C for 30 s (35 cycles)	Fast SYBR^TM^ Green Master Mix	[31]
*bla_TEM_* (113 bp)	bla_TEM_-F (TTCCTGTTTTTGCTCACCCAG) bla_TEM_-R (CTCAAGGATCTTACCGCTGTTG)	pNORM (digested)	96	95 °C for 10 min (1 cycle); 95 °C for 15 s, and 60 °C for 1 min (40 cycles)	SYBR^®^ Select Master Mix	[32]

**Table 2:** Growth parameters (average ± standard deviation, n = 3) determined for each PBR. The specific growth rate (average ± standard deviation, n = 3) is only shown for the PBRs whose data fit the modified Gompertz model. Values with a different letter and within the same column are statistically different (*p* < 0.05).

PBR	MCC_max_ (Cells mL^−1^)	P_X,max_ (Cells mL^−1^ d^−1^)	P_X,avg_ (Cells mL^−1^ d^−1^)	µ (d^−1^)	R^2^	RMSE
MBS	(1.2 ± 0.2) × 10^7 a^	(3.3 ± 0.6) × 10^6 a^	(1.2 ± 0.3) × 10^6 a^	0.651 ± 0.155 ^a^	0.990	0.046
C+	(9.7 ± 0.1) × 10^6 a^	(2.3 ± 0.1) × 10^6 b^	(6.4 ± 0.2) × 10^5 b^	0.470 ± 0.131 ^a^	0.931	0.030
C−	(3.3 ± 0.2) × 10^6 c^	(2.3 ± 0.2) × 10^6 b^	(5.4 ± 0.3) × 10^5 b^	1.442 ± 0.451 ^b^	0.944	1.247
DC−	(7.8 ± 1.6) × 10^4 d^	(3.1 ± 3.1) × 10^4 c^	(−2.6 ± 2.6) × 10^3 c^	-	-	-

MCC_max_: maximum value of microalgal cell concentration; PBR: photobioreactor; P_X,avg_: average productivity; P_X,max_: maximum productivity; R^2^: coefficient of determination; RMSE: root mean square error; µ: specific growth rate.

**Table 3:** Nitrogen and phosphorus initial concentration, removal parameters, and kinetic constant obtained by the modified Gompertz model for each PBR. Values are presented as the average ± standard deviation (n = 3). For each nutrient, values with a different letter and within the same column are statistically different (*p* < 0.05).

Nutrient	EU Legislation Limit	PBR	S_0_ (mg L^−1^)	MR (mg L^−1^)	RR (mg L^−1^ d^−1^)	RE (%)	Y_X/S_ (g_DW_ g_nutrient_^−1^)	k (d^−1^)	R^2^	RMSE (mg L^−1^)
NH_4_^+^-N	15 mg L^−1^	MBS	57.47 ± 9.14 ^a^	54.30 ± 7.45 ^a^	9.05 ± 1.24	95 ± 2 ^a^	7.73 ± 4.42 ^a,b^	0.806 ± 0.260 ^a^	0.918	1.228
		C+	41.00 ± 0.28 ^b^	33.90 ± 0.01 ^b^	5.65 ± 0.01	83 ± 1 ^b^	7.68 ± 0.13 ^b^	0.872 ± 0.157 ^a^	0.963	1.190
		C−	65.00 ± 1.95 ^a^	38.77 ± 1.71 ^b^	6.46 ± 0.28	60 ± 1 ^c^	3.31 ± 0.32 ^a^	-	-	
		DC−	65.00 ± 1.95 ^a^	3.83 ± 2.06 ^c^	0.64 ± 0.34	6 ± 3 ^d^	−0.27 ± 0.39 ^c^	-	-	
PO_4_^3−^-P	2 mg L^−1^	MBS	5.08 ± 0.43 ^a^	4.75 ± 0.34 ^a^	0.79 ± 0.06	94 ± 1 ^a^	85.94 ± 28.61 ^a^	3.829 ± 1.330 ^a^	0.993	0.048
		C+	2.32 ± 0.08 ^b^	1.94 ± 0.07 ^b^	0.32 ± 0.01	84 ± 1 ^b^	134.57 ± 4.40 ^b^	5.939 ± 4.231 ^a^	0.984	0.040
		C−	1.25 ± 0.02 ^c^	1.17 ± 0.02 ^c^	0.19 ± 0.01	94 ± 1 ^a^	109.54 ± 4.24 ^a,b^	-	-	
		DC−	1.25 ± 0.02 ^c^	−0.13 ± 0.03 ^d^	−0.02 ± 0.01	−10 ± 2 ^c^	4.25 ± 4.24 ^c^	-	-	

DW: biomass dry weight; k: uptake rate; MR: mass removal; PBR: photobioreactor; R^2^: coefficient of determination; RE: removal efficiency; RMSE: root mean square error; S_0_: Initial concentration; Y_X/S_: yield of biomass based on nutrient consumption.

**Table 4:** Initial abundance (A_0_) of *intl*1 and resistance genes (*sul*1 and *bla_TEM_*) and percentage of the reduction of gene prevalence (RGP) determined for each PBR (n = 2).

PBR	*intI*1		*sul*1		*bla_TEM_*	
	A_0_ (Gene Copy Number 100 mL^−1^)	RGP (%)	A_0_ (Gene Copy Number 100 mL^−1^)	RGP (%)	A_0_ (Gene Copy Number 100 mL^−1^)	RGP (%)
MBS	(4.0 ± 0.5) × 10^7^	93 ± 2	(4.3 ± 1.1) × 10^7^	42 ± 8	(1.1 ± 0.4) × 10^2^	91.7 ± 2.5
C+	(9.0 ± 1.0) × 10^7^	89 ± 1	(8.0 ± 0.3) × 10^7^	46 ± 4	(2.8 ± 0.2) × 10^1^	31.0 ± 2.3
C−	(3.8 ± 0.2) × 10^7^	1 ± 5	(5.1 ± 0.1) × 10^7^	25 ± 138	(1.8 ± 0.1) × 10^3^	58.6 ± 2.5
DC−	(3.8 ± 0.2) × 10^7^	−35 ± 3	(5.1 ± 0.1) × 10^7^	−1 ± 6	(1.8 ± 0.1) × 10^3^	48.5 ± 6.3

**Table 5:** Prevalence of *intl*1 and resistance genes (*sul*1 and *bla_TEM_*) on day 8 of storage and percentage reduction of gene prevalence (RGP) from day 0 to St_8_ determined for each PBR (n = 2).

PBR	*intI*1		*sul*1		*bla_TEM_*	
	Prevalence of St_8_ (GA/16S rRNA)	RGP (%)	Prevalence of St_8_ (GA/16S rRNA)	RGP (%)	Prevalence of St_8_ (GA/16S rRNA)	RGP (%)
MBS	(2.5 ± 1.0) × 10^−3^	91 ± 5	(9.5 ± 7.3) × 10^−3^	72 ± 13	(1.3 ± 0.2) × 10^−9^	98.2 ± 0.6
C+	(3.8 ± 0.1) × 10^−3^	90 ± 1	(6.1 ± 0.1) × 10^−3^	82 ± 1	(1.2 ± 0.1) × 10^−9^	89.8 ± 1.5
C−	(4.1 ± 0.6) × 10^−3^	76 ± 4	(1.2 ± 0.3) × 10^−2^	50 ± 9	(2.6 ± 0.2) × 10^−8^	96.8 ± 0.5
DC−	(1.2 ± 0.1) × 10^−1^	−569 ± 31	(3.1 ± 0.1) × 10^−2^	−32 ± 13	(5.8 ± 0.1) × 10^−8^	93.1 ± 0.7

## Data Availability

The raw data supporting the conclusions of this article will be made available by the authors on request.

## Supplementary Materials

The following supporting information can be downloaded at: https://www.mdpi.com/article/10.3390/microorganisms12071421/s1. Figure S1: Experimental setup (from left to right: C+, MBS triplicates, C−, and DC−); Figure S2: Microscopic observation (400×) of microalgal species detected in the MBS at day 0. Each square has an area of 0.0025 mm^2^; Figure S3: pH variation (average ± standard deviation) over time (d) for each PBR:  MBS;  C+;  C−; and  DC−. St_3:_ day 3 of storage; St_8_: day 8 of storage; Figure S4: Pearson correlation of all the analysed variables obtained for each PBR in E-II: MBS, C+, C−, and DC−. The * symbol discriminates correlations with *p* < 0.05; Table S1: Composition of the modified OECD microalgae culture medium.; Table S2: Calibration curves’ parameters used in this study; Table S3: Average decrease rate of gene prevalence (gene copy number/16S rRNA copy number/d) of *intl*1 and resistance genes (sul1 and bla_TEM_) during the cultivation period determined for each PBR. For each gene, values (average ± standard deviation, n = 6) with a different superscript letter are statistically different (*p* < 0.05).

## Abbreviations

ARG	Antibiotic-resistant gene
C−	Negative control
C+	Positive control
CFU	Colony-forming unit
COD	Chemical oxygen demand
DC−	Dark negative control
DNA	Deoxyribonucleic acid
DO	Dissolved oxygen
DW	Dry weight
EPS	Extracellular polymeric substances
HA	Humic acid
LOD	Limit of detection
LOQ	Limit of quantification
MBS	Microalgae-bacteria system
NH_4_^+^-N	Ammonium-nitrogen
NO_3_^−^-N	Nitrate-nitrogen
OECD	Organisation for Economic Cooperation and Development
PBR	Photobioreactor
PO_4_^3−^-P	Phosphate-phosphorus
qPCR	Quantitative polymerase chain reaction
rRNA	Ribosomal ribonucleic acid
UWW	Urban wastewater
UWWTP	Urban wastewater treatment plant
