# Supplementary material for: Native Microalgae-Bacteria Consortia: A Sustainable Approach for Effective Urban Wastewater Bioremediation and Disinfection
Source: Microorganisms. 2024 Jul 12;12(7):1421. doi: 10.3390/microorganisms12071421 (PMC11278754; doi:10.3390/microorganisms12071421)
Supplement: Supplementary file 1 [file microorganisms-12-01421-s001.zip › microorganisms-3092069-supplementary.pdf]

Supplementary material

Table S1. Composition of the modified OECD microalgae culture medium.

Solution	Reagent	Concentration in the media (mg L^{-1})
A	$NaNO_3$	250
	$MgCl_2 \cdot 6\ H_2O$	12
	$CaCl_2 \cdot 2\ H_2O$	18
	$MgSO_4 \cdot 7\ H_2O$	15
	KH_2PO_4	45
B	$FeCl_3 \cdot 6\ H_2O$	0.08
	$Na_2EDTA \cdot 2\ H_2O$	0.1
C	H_3BO_3	0.185
	$MnCl_2 \cdot 4\ H_2O$	0.415
	$ZnCl_2$	3×10^{-3}
	$CoCl_2 \cdot 6\ H_2O$	1.5×10^{-3}
	$CuCl_2 \cdot 2\ H_2O$	0.01×10^{-3}
	$Na_2MoO_4 \cdot 2\ H_2O$	7×10^{-3}
D	$NaHCO_3$	500

Table S2. Calibration curves' parameters used in this study.

x	y	$a \pm s_a$	$b \pm s_b$	R^2	LOD_x	LOQ_x	n
Biomass concentration (g_{DW} mL^{-1})	MCC (cells mL^{-1})	$(2.46 \pm 0.31) \times 10^{10}$	$(-3.12 \pm 1.51) \times 10^{6}$	0.969	1.84×10^{-4}	6.15×10^{-4}	4
HA (mg L^{-1})	$Abs_{532\ nm}$	0.029 ± 0.001	0.004 ± 0.033	0.994	3.40	11.35	6
COD (mg L^{-1})	$Abs_{420\ nm}$	$(2.73 \pm 0.11) \times 10^{-3}$	(1.60 ± 0.59) 10^{-2}	0.993	6.44	21.47	7
PO_4-P (mg L^{-1})	$Abs_{820\ nm}$	0.184 ± 0.002	0.004 ± 0.003	1.000	0.06	0.19	7
NO_3-N (mg L^{-1})	$Abs_{410\ nm}$	$(5.10 \pm 0.08) \times 10^{-2}$	$(1.76 \pm 0.44) \times 10^{-2}$	0.998	0.26	0.87	8

a: slope of the calibration curve; Abs: absorbance; b: intercept; COD: chemical oxygen demand; DW: dry weight; LOD_x: limit of detection; LOQ_x: limit of quantification; MCC: microalgal cell concentration; n: number of standards; NO_3-N: nitrate; PO_4-P: phosphate; R^2: coefficient of determination; s_a: standard deviation of the slope; s_b: standard deviation of the intercept.

Table S3. Average decrease rate of gene prevalence (gene copy number/16S rRNA copy number/d) of *intI1* and resistance genes (*sul1* and *bla*$_{TEM}$) during the cultivation period determined for each PBR. For each gene, values (average ± standard deviation, n = 6) with a different superscript letter are statistically different ($p < 0.05$).

PBR	*intI1*	*sul1*	*bla*$_{TEM}$
MBS	$(4.8 \pm 1.2) \times 10^{-3}$ [a]	$(2.4 \pm 1.2) \times 10^{-3}$ [a]	$(1.2 \pm 0.2) \times 10^{-8}$ [a]
C+	$(5.5 \pm 1.7) \times 10^{-3}$ [a]	$(2.5 \pm 1.0) \times 10^{-3}$ [a]	$(6.0 \pm 3.7) \times 10^{-10}$ [a]
C-	$(0.4 \pm 7.1) \times 10^{-4}$ [b]	$(1.0 \pm 0.3) \times 10^{-3}$ [b]	$(8.2 \pm 3.6) \times 10^{-8}$ [b]
DC-	$(-6.9 \pm 8.6) \times 10^{-4}$ [b]	$(-0.4 \pm 2.6) \times 10^{-4}$ [b]	$(6.7 \pm 1.8) \times 10^{-8}$ [b]

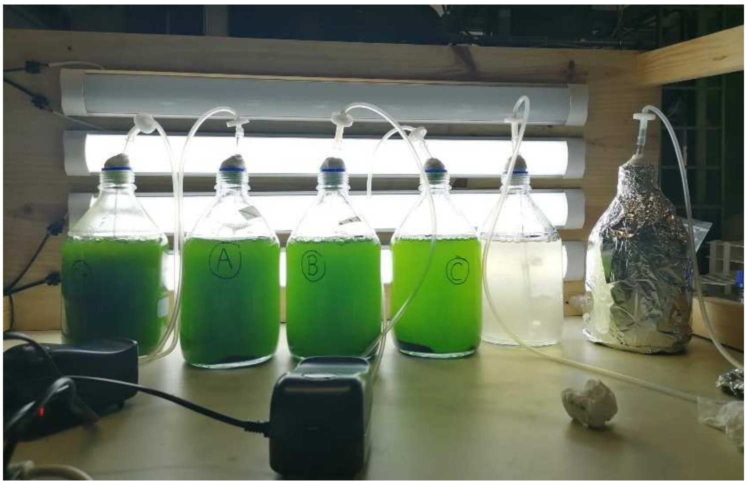

Figure S1. Experimental setup (from left to right: C+, MBS triplicates, C- and DC-).

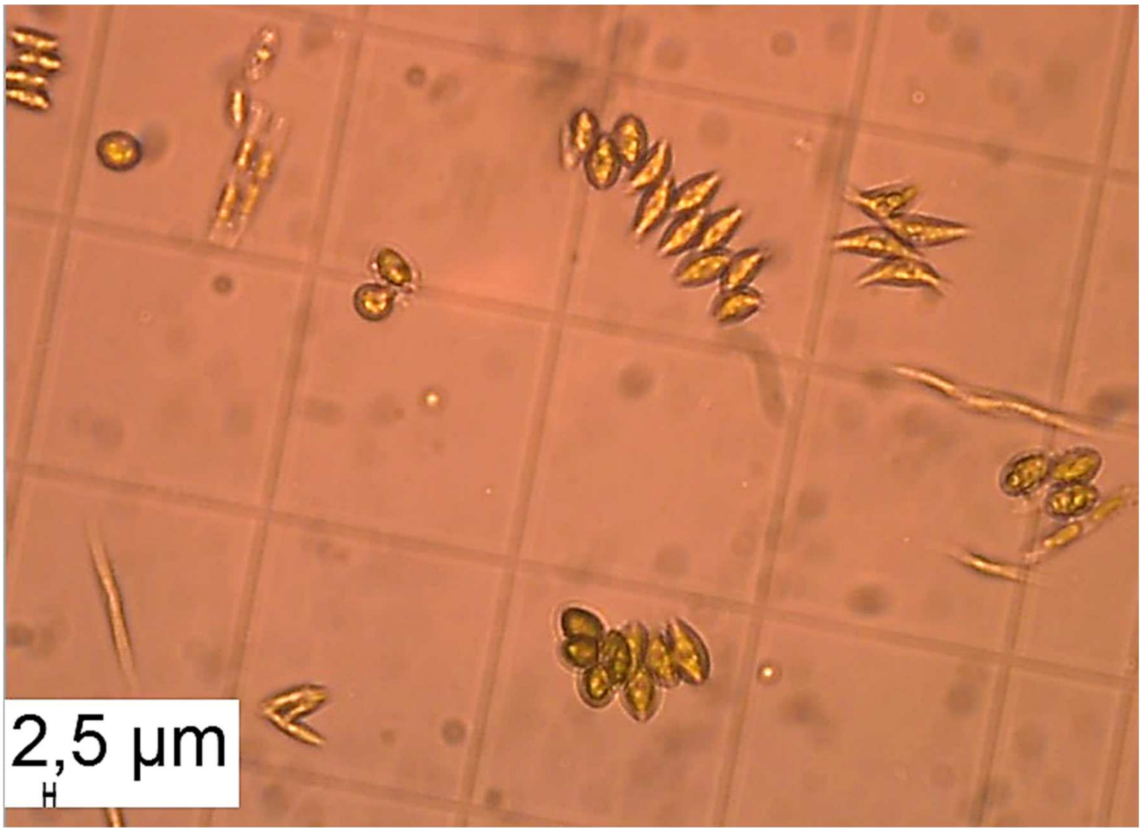

Figure S2. Microscopic observation (400×) of microalgal species detected in the MBS at day 0. Each square has an area of 0.0025 mm^2.

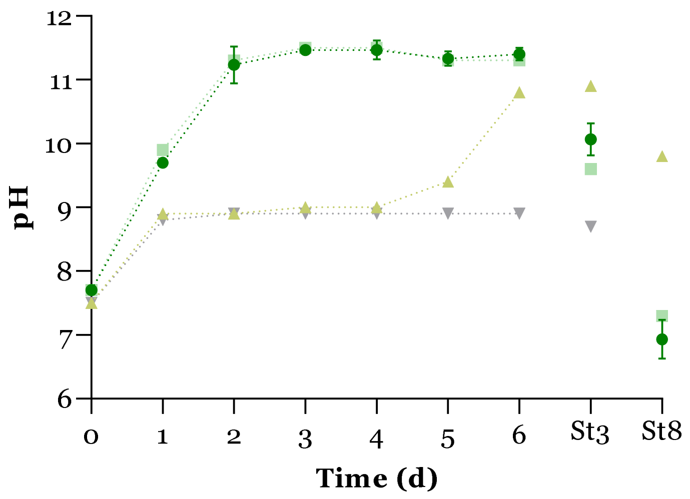

Figure S3. pH variation (average ± standard deviation) over time (d) for each PBR: ● MBS; ■ C+; ▲ C-; and ▼ DC-. St3: day 3 of storage; St8: day 8 of storage.

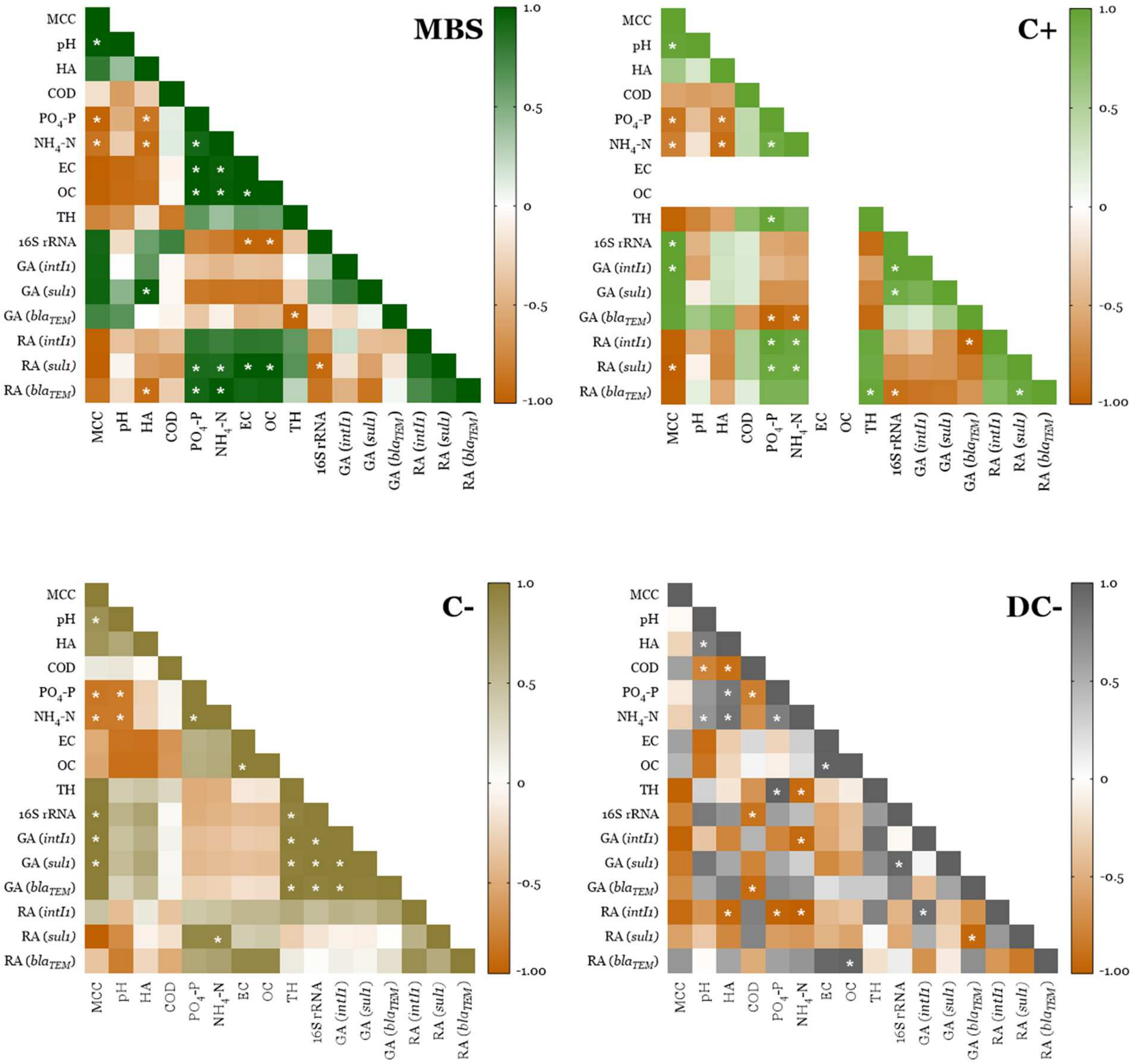

Figure S4. Pearson correlation of all the analyzed variables obtained for each PBR in E-II: MBS, C+, C-, and DC-. The * symbol discriminates correlations with $p < 0.05$.

COD: chemical oxygen demand concentration; EC: *E. coli* abundance; GA: gene abundance; HA: humic acid concentration; MCC: microalgal cell concentration; NH_4-N: ammonium-nitrogen concentration; OC: other coliforms (except *E. coli*) abundance; PO_4-P: phosphate-phosphorus concentration; RA: relative abundance (prevalence); TH: total heterotrophs concentration.
